# Spontaneous early pregnancy with very low levels of progesterone during controlled ovarian stimulation-A case report

**DOI:** 10.5935/1518-0557.20210115

**Published:** 2022

**Authors:** Abha Majumdar, Ruchi Joshi

**Affiliations:** 1 Sir Ganga Ram Hospital, New Delhi, India

**Keywords:** progesterone, pregnancy, ovarian hyperstimulation, *in vitro* fertilization, low progesterone during early pregnancy

## Abstract

One of the most widely accepted axioms of reproductive biology is that pregnancy requires the sole support of progesterone, without which pregnancy cannot be established or maintained. We report a rare case of ongoing third trimester pregnancy in a 41-year-old woman, where early gestational period was maintained despite extremely low progesterone levels of <1 ng/ml, and was discovered during controlled ovarian hyperstimulation (COS) for *in vitro* fertilization (IVF). She was started on ovarian stimulation (OS) with gonadotrophins after a withdrawal bleed during lactational amenorrhea. Baseline investigations on day 2 of the menstruation confirmed low serum estradiol and progesterone levels (<1 ng/ml). After 5 days of stimulation, on ultrasound scan, a sac-like structure was seen in the uterine cavity. Beta hCG levels were measured and confirmed the presence of early pregnancy despite progesterone levels below 1 ng/ml. COS was stopped, and progesterone support was started. Subsequent scan confirmed live intrauterine pregnancy and the fetus is currently growing uneventfully at 31 weeks of gestation (at the time of writing this report).

## INTRODUCTION

Traditionally, we have believed that progesterone is essential for both conception and maintenance of pregnancy. However, there are very few cases about continuing pregnancy with extremely low levels of progesterone in early gestation. We report an unusual, case in which we started ovarian stimulation (OS) for in vitro fertilization (IVF) with an antagonist protocol, after withdrawal bleeding in a 41 year old woman with lactational amenorrhea. On day 2 of the menstrual cycle, ultrasound scan of the uterus and serum hormone levels were within normal limits, showing low serum estrogen and progesterone levels <1 ng/ml. After 5 days of OS, the ultrasound scan showed a gestational sac-like structure in the uterine cavity and pregnancy was confirmed by beta hCG levels. The progesterone levels continued to be lower than 1 ng/ml, but the estradiol levels started to rise. This pregnancy could have been achieved only by an ovulation, which occurred 3 weeks prior to the day-6 scan, following ovarian stimulation, yet progesterone levels were less than 1 ng/ml till the appearance of the intrauterine sac.

## CASE REPORT

A 41-year-old woman came to our tertiary care reproductive center during her lactational amenorrhea to start with a second IVF cycle. She had come to us 2 years ago with primary infertility, when she underwent her 1^st^ cycle, in which 2 oocytes were retrieved and 1 day-3 embryo was formed, fresh embryo transfer was done and she conceived her first child who is now 16 months of age.

**Figure 1:**
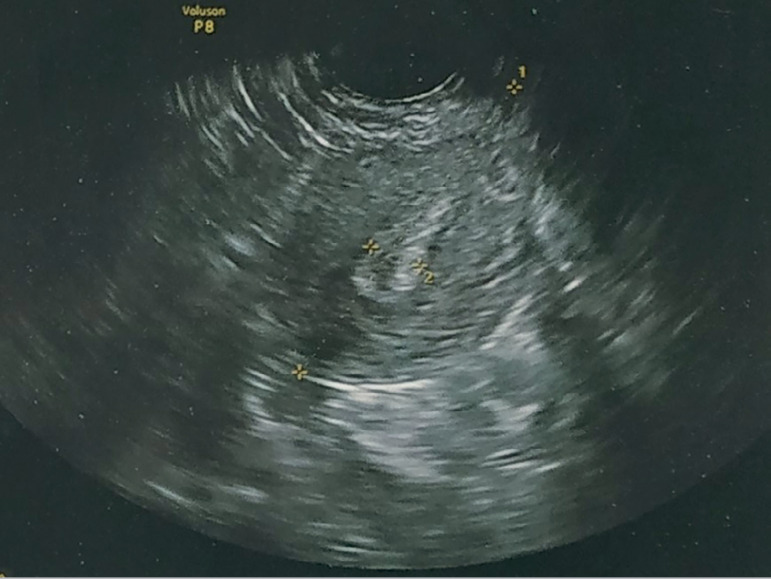
Baseline ultrasound before OS.

**Figure 2:**
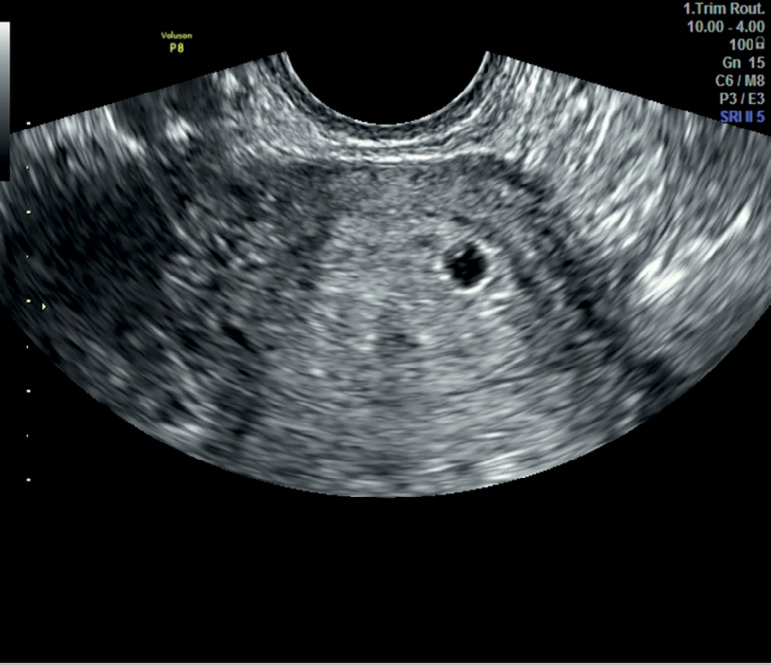
Ultrasound with early gestation sac.

After all preliminary investigations she registered for IVF again. She had a low ovarian reserve indicated by an AMH value of 0.22 ng/ml, and an antral follicle count of 6 in both ovaries, with endometrial thickness of 7.2 mm (Figure 1). We decided to take her for controlled ovarian hyperstimulation (COS) with the antagonist protocol, and administrated medroxy progesterone acetate (MPA) for withdrawal bleeding, which happened within a week of the last dose. Her ultrasound revealed silent ovaries with endometrial thickness (ET) of 6.9 mm. Her baseline hormone assay on day2 showed: (FSH)-9.52 mIU/mL; (LH), 6.59 mIU/mL; E2-64 mIU/ml; P4 was 0.32 ng/ml and COS was started with the Injection of Follicular Stimulating Hormone 300U s/c (Bharat serum) and Injection of Luteinising hormone 75 U (Merck) subcutaneously over the next 5 days. As per routine protocol, we repeated her ultrasound and blood investigations on day 6 of the OS. Serum assessment of hormones revealed LH 5.86mIU/ml, E2 156mIU/ml and serum P4 0.08 ng/ml. Her pelvic ultrasound showed 4 follicles in both ovaries, but a small sac-like structure, measuring 3 mm within the uterine cavity (Figure 2). Beta hCG levels were measured and were found to be 717.69 mIU/ml. Beta hCG level was tested on her day-2 blood sample (which is preserved in our center as routine till the conclusion of COS), and they came out to be 285 mIU/ml. We stopped with the gonadotropins and she was started on vaginal progesterone 400mg twice a day. It is imperative to note that she must have ovulated 3 weeks prior to the day-6 scan during OS for IVF. However, her progesterone was still extremely low when this pregnancy was first detected.

After 14 days we repeated her ultrasound and she had single live intrauterine fetus of 6 weeks with cardiac activity. Her serum progesterone levels were 28 ng/ml. Her 12-week ultrasound scan with double markers, non-invasive prenatal testing (NIPT) and level 2 scan were also normal. Currently she is doing well without any complications into the 31^st^ week of gestational age.

## DISCUSSION

The most important point to be discussed in this case is the continuation of pregnancy despite extremely low progesterone levels of less than 1ng/ml. Maintenance of pregnancy requires production of progesterone from the corpus luteum after ovulation and during the early first trimester, until placental function is established. Removal of the corpus luteum prior to the development of adequate placental function results in spontaneous pregnancy loss (Csapo *et al*., 1972). Luteal phase is the period between ovulation and either establishment of pregnancy or onset of the next menses (Fatemi *et al*., 2007). Embryonic implantation occurs during the implantation window, when a perfect synchronization of embryonic and endometrial signals is essential. Progesterone is mandatory for the secretory transformation of the endometrium, which enables implantation as well as maintenance of early pregnancy (Daya, 2009). Following implantation, the developing blastocyst secretes human chorionic gonadotrophin (HCG). The HCG maintains corpus luteum function (Penzias, 2002). To confirm ovulation, values at midluteal phase should be at least 6.5 ng/ml and preferably >10 ng/ml (Speroff & Fritz, 2005). In our case it was novel as to how the pregnancy continued despite such low levels of serum progesterone.

Upon reviewing the literature, we found a similar case reported by Takenaka *et al*. (2016) of unnoticed pregnancy that was maintained despite low progesterone levels (0.4 ng/ml), detected 4 days after oocyte retrieval.

## CONCLUSION

This report describes a rare case of early pregnancy with menstrual bleeding, in which endometrial thickness, E2 and P levels were inadequate by all accepted criteria, and the gestational sac was visible with a progesterone level of <1 ng/ml. Despite negligible progesterone levels, the pregnancy continued till the third trimester. There may be other factors which support pregnancy despite temporary lack of progesterone, and which mandates further research.
